# Comparison of hemodialysis and peritoneal dialysis patients’ dietary behaviors

**DOI:** 10.1186/s12882-020-01744-6

**Published:** 2020-03-10

**Authors:** Seon-Mi Kim, Byung Chin Kang, Hyun-Jung Kim, Min-Sook Kyung, Hyung Jung Oh, Jung-Hyun Kim, Oran Kwon, Dong-Ryeol Ryu

**Affiliations:** 1 Department of Internal Medicine, Graduate School, Ewha Womans University, Seoul, Korea; 2 Department of Nutrition, Ewha Womans University, Seoul Hospital, Seoul, Korea; 3 Graduate School of Converging Clinical and Public Health, Ewha Womans University, Seoul, Korea; 4 College of Nursing, Ewha Womans University, Seoul, Korea; 5 Graduate School, Yonsei University, Seoul, Korea; 6 Ewha Institute of Convergence Medicine, Ewha Womans University, Mokdong Hospital, Seoul, Korea; 7 Research Institute for Human Health Information, Ewha Womans University, Mokdong Hospital, Seoul, Korea; 8 Department of Home Economics Education, Pai Chai University, Daejeon, Korea; 9 Department of Nutritional Science and Food Management, Ewha Womans University, Seoul, Korea; 10 Department of Internal Medicine, School of Medicine, Ewha Womans University, 260, Gonghang-daero, Seoul, Gangseo-gu Korea

**Keywords:** Dietary behavior, Nutritional intake, Peritoneal dialysis, Hemodialysis

## Abstract

**Background:**

Nutritional factors are associated with high mortality and morbidity in dialysis patients, and protein-energy wasting is regarded as an important one. The modality of dialysis may affect patients’ dietary behavior and nutritional status, but no study has compared the dietary behavior, nutrient intake, and nutritional adequacy of hemodialysis (HD) and peritoneal dialysis (PD) patients.

**Methods:**

From December 2016 to May 2017, a dietary behavior survey and Semi-quantitative Food Frequency Questionnaire (Semi-FFQ) were conducted on 30 HD patients and 30 PD patients in Ewha Womans University Mokdong Hospital, and laboratory parameters were obtained. The results of prevalent HD and PD patients were then compared.

**Results:**

The mean age of HD patients was higher than that of PD patients; HD: 58.5 ± 9.1 years, PD: 49.3 ± 9.7 years (*p* = 0.001). In the dietary behavior survey, HD patients showed more appropriate dietary behavior patterns overall than PD patients. In the dietary intake analysis with the Semi-FFQ, energy intake was significantly lower in the PD group than in the HD group due to the lower intake of carbohydrates, fat, and protein. A comparison of nutrient intake-to-recommended allowance ratio between the HD and PD groups revealed that the HD group showed higher nutrient intake than the PD group. Serum albumin and potassium levels were significantly higher in HD than in PD patients.

**Conclusion:**

According to this study, the dietary behavior and nutritional intake of prevalent PD patients were worse than those of HD patients.

## Background

Patients reaching end-stage renal disease (ESRD) must decide between hemodialysis (HD) and peritoneal dialysis (PD) unless they are offered the opportunity to undergo kidney transplantation. Since the prognosis of ESRD according to dialysis modality is not significantly different, dialysis modality is mainly chosen after full consideration of both medical and non-medical factors, including patient preference and social factors [1].

The health status of dialysis patients is likely to be very poor; it was found to be as low as 54% of that of healthy individuals in Korea [2]. Among the factors associated with high mortality and morbidity in dialysis patients, protein-energy wasting (PEW) is regarded as an important one. The prevalence of PEW among maintenance HD patients varies from 18 to 56% [3, 4]. PEW results from reduced dietary intake, inflammation, resistance to anabolic hormones, loss of nutrients during dialysis, gastrointestinal disturbance-induced medication (e.g., phosphate binders and iron supplements), comorbid conditions due to uremia, decrease physical activity, and the breakdown of muscle protein induced by metabolic acidosis [5]. Especially, lack of exercise and muscle weakness by measures including hand grip strength, mid-arm circumference relates to PEW and mortality to Chronic kidney disease (CKD) patients. In addition, PD patients are in a unique situation compared to HD patients: they experience similar net weekly nitrogen losses to HD patients despite protein loss of 6 to 8 g/day to peritoneal fluid [6] and on average 400 kcal of energy intake from obligatory glucose absorption [7]. Unlike HD, PD is typically performed daily; thus, the body does not build up as much potassium, sodium, and fluid. Diet has low restriction due to the daily sessions of peritoneal dialysis. Also, residual renal function in patients with peritoneal dialysis increases small solute clearance and intake of actual dietary nutrients. Hence, most PD patients are recommended a diet that is more liberal than that of HD patients [8]. According to this dissimilarity in the nature of dialysis, dietary counseling usually differs for HD and PD.

Consequently, HD and PD patients may have different diet patterns and differ in nutritional status. However, although a few studies have compared the nutritional status of HD and PD patients [9–11], no study has assessed and compared nutrient intake by the Semi-quantitative Food Frequency Questionnaire (Semi-FFQ) and dietary behavior according to dialysis modality. Thus, in this study, we investigated whether prevalent HD and PD patients’ dietary behavior and nutrient intake differed.

## Methods

### Subjects

All eligible HD and PD patients of Ewha Womans University Mokdong Hospital, who voluntarily participated with written informed consent, were enrolled between December 2016 and May 2017. The number of original patient cohort was 93 patients for HD group and 67 patients for PD group. Included were outpatients who had been undergoing dialysis treatment for at least 3 months and agreed to participate in this study voluntarily. We excluded patients who were younger than 18 years of age, had a plan for renal transplantation within 3 months, had a life expectancy of less than 3 months, had distinct edema observed by the investigators, acute disease phase leading to admission, hybrid dialysis type using both of hemodialysis and peritoneal dialysis, and cognitive impairments who are unable to complete questionnaires. The study was performed in accordance with the Declaration of Helsinki and was approved by the Institutional Review Board of Ewha Womans University Medical Center (EUMC 2016–09–039-001).

### Data collection

Demographic and clinical data were recorded at the time of the study by a self-report survey and included sex, age, duration of dialysis, comorbidities, marital status, work status, family support, smoking, alcohol, and medication (including anti-hypertensive agents, phosphate binders, and supplemental iron). Patients with medications that were maintained for at least 2 months before enrollment were marked as having maintenance medication.

Decreased physical activity plays an important role in etiology of PEW, so we analyzed body mass index (BMI), mid-arm circumference (MAC), and hand grip strength (HGS). HGS was measured in the hand without vascular access for HD patients after hemodialysis and in the dominant hand for PD patients. Among HD patients, 24 patients of HD had arteriovenous fistula, and 8 patients (20%) had arteriovenous graft. HGS was measured using a digital hand dynamometer (Digital grip strength dynamometer, T.K.K 5401, Takei Scientific Instruments Co., Ltd., Tokyo, Japan).

Nutritional status was determined using the 7-point subjective global assessment (SGA), scale consisting of two categories: medical history and physical examination. The medical history section included weight change, dietary intake, gastrointestinal symptoms, functional capacity, and disease and comorbidity data. The physical examination section included loss of subcutaneous fat, muscle wasting, and edema. The trained investigators rated each item from 1 to 7 and decided the overall SGA score. Based on the overall SGA score, the patients were categorized into three groups: well nourished (normal) = SGA score of 6–7, mildly to moderately malnourished = SGA score of 3–5, or severely malnourished = SGA score of 1–2 [12].

In Republic of Korea, insurance-covered nutritional education programs are available for CKD patients: at initial diagnosis; and when dialysis is started. However, the program is not mandatory, and the patients are not aware of the program. Through a questionnaire, Presence of diet education prior to dialysis initiation was also recorded.

### Dietary behaviors

A questionnaire consisting of 10 items on the patient’s appropriate or inappropriate dietary behaviors was used, which was a modified version of a questionnaire for dialysis patients from a previous study [12]. The questionnaire responses were divided into three categories according to the fulfillment frequency on each question: 0 to 2 days, 3 to 5 days, or 6 to 7 days during the week. For questions in which the number of days of appropriate diet activity for health was asked, we gave the scores 0, 1, and 2 for each response in order. In contrast, for questions in which the number of days of inappropriate diet activity for health was asked, we gave the scores 2, 1, and 0 for each response in order. Therefore, higher values represented better dietary behavior. The survey was conducted twice with an interval of more than 1 week, and the average values of the survey were used.

### Semi- FFQ

The Semi-FFQ is the most commonly used questionnaire in epidemiological studies to assess long-term nutritional exposure [13]. The FFQ examines the intake frequency during a standard period as well as the amount of the listed food categories. Recently, the Semi-FFQ has been used for the assessment of nutritional intake in dialysis patients [14]. We designed the semi-FFQ for Korean dialysis patient and reported that this questionnaire would be a reliable tool for the assessment of the HD patients’ nutrient intake along with the 7-day dietary record [15]. The correlation coefficients were higher for foods consumed daily, such as steamed rice, meat and chicken, bean, egg, milk, coffee and alcohol, while those were lower in foods eaten rarely. A Semi-FFQ including 47 food items which is reflected dialysis patients’ diet was presented (Table S1), and the food items listed in the questionnaire were based on the Korean Health and Nutrition Survey. The Semi-FFQ consisted of groups of grains (rice, bread, and rice cake), meat, fish, beans, eggs, potatoes, dairy products, vegetables, and fruits classified as having low, medium, and high potassium content. The survey was conducted twice with an interval of more than 1 week, and the mean values were used.

### Laboratory parameters

The results of the most recent blood test within 1 month from the time of the survey were collected retrospectively. Blood samples were taken mid-week before the HD session for HD patients and on the outpatient visit day for PD patients. We measured the concentrations of serum albumin, blood urea nitrogen (BUN), creatinine, hemoglobin, hematocrit, sodium, phosphorus, calcium, potassium, and serum glucose.

### Statistical analysis

The results of the food intake amount and the frequency were analyzed by applying the nutrient evaluation program CAN-Pro 4.0. We analyzed the intake amounts for 20 nutrients, using the ninth revision of the National Standard Food Composition Table published by the Rural Development Administration in Korea [16]. In addition, the intake of each nutrient was compared to the recommended nutrient reference value [17].

The general characteristics, dietary behaviors, laboratory parameters, and Semi-FFQ scores of the subjects were presented as mean and standard deviation or N (%), and *p*-value < 0.05 was considered significant. For the comparison of the characteristics of the HD and PD groups, Student’s t-test and the chi-square test were used. Statistical analyses were conducted using the SPSS statistical package for Windows Ver. 23.0 (SPSS, Inc., Chicago, IL, USA).

## Results

### General characteristics

The clinical characteristics of subjects and dialysis modality are shown in Table 1 and Table S2, respectively. The mean age of HD patients was higher than that of PD patients; HD: 58.5 ± 9.1 years, PD: 49.3 ± 9.7 years (*p* = 0.001). The duration of dialysis was not significantly different between the two groups. We also compared the anthropometric value of each group. The mean BMI of PD patients was higher than that of HD patients; HD: 23.1 ± 4.0 kg/m^2^, PD: 25.6 ± 4.2 kg/m^2^ (*p* = 0.02). HGS was higher in the PD group; HD: 20.6 ± 9.3kg, PD: 25.6 ± 8.1 kg (*p* = 0.034). There was no significant difference in pre-dialysis nutrition education between the two groups.

**Table 1:** General characteristics of patients

Variables	HD (*n* = 30)	PD(n = 30)	*p*-value	HD (n = 30)			PD (n = 30)		
				Male (*n* = 19)	Female (*n* = 11)	*p*-value	Male (*n* = 16)	Female (*n* = 14)	*p*-value
Age (years)	58.5 ± 9.1	49.3 ± 9.7	0.001^**^	57.6 ± 10.6	60.1 ± 5.6	0.405	51.8 ± 9.7	46.6 ± 9.3	0.148
Duration of Dialysis (months)	50.9 ± 52.3	63.4 ± 56.4	0.377	48.2 ± 59.6	55.7 ± 38.7	0.709	53.6 ± 56.5	74.6 ± 56.3	0.317
Comorbidities									
Yes	30 (100.0)	29 (96.67)	0.313	19 (100.0)	13 (100.0)	NA	16 (94.1)	13 (100.0)	0.374
No	0 (0.0)	1 (3.33)		0 (0.0)	0 (0.0)		1 (5.9)	0 (0.0)	
Marital status									
Yes	24 (80.0)	20 (66.7)	0.076	14 (73.7)	10 (90.9)	0.265	11 (64.7)	9 (69.2)	0.184
No	6 (20.0)	10 (33.3)		5 (26.3)	1 (9.1)		6 (35.3)	4 (30.8)	
Work status									
Yes	6 (20.0)	16 (53.3)	0.007^**^	6 (31.6)	0 (0.0)	0.037^*^	11 (64.7)	5 (38.5)	0.153
No	24 (80.0)	14 (46.7)		13 (68.4)	11 (100.0)		6 (35.3)	8 (61.5)	
Family support									
Yes	26 (86.7)	26 (86.7)	1.000	16 (84.2)	10 (52.6)	0.862	15 (88.2)	11 (84.6)	0.794
No	3 (10.0)	3 (10.0)		2 (10.5)	1 (5.3)		2 (11.6)	1 (7.7)	
No response	1 (3.3)	1 (3.3)		1 (5.3)	0 (0.0)		0 (0.0)	1 (7.7)	
Smoking									
Yes	2 (6.7)	3 (10.0)	0.640	2 (10.5)	0 (0.0)	0.265	3 (17.7)	0 (0.0)	0.110
No	28 (93.3)	27 (90.0)		17 (89.5)	11 (100.0)		14 (82.4)	13 (100.0)	
Alcohol									
Yes	0 (0.0)	2 (6.7)	0.150	0 (0.0)	0 (0.0)	NA	1 (5.9)	1 (7.7)	0.844
No	30 (100.0)	28 (93.3)		19 (100.0)	11 (100.0)		16 (94.1)	12 (92.3)	
Taking any medication									
Yes	29 (96.7)	29 (96.7)	NA	18 (94.7)	11 (100.0)	NA	16 (94.1)	13 (100.0)	NA
No	0 (0.0)	1 (5.3)		0 (0.0)	0 (0.0)		1 (5.9)	0 (0.0)	
No response	1 (5.3)	0 (0.0)		1 (5.3)	0 (0.0)		0 (0.0)	0 (0.0)	
Anti-hypertensive									
Yes	23 (76.7)	27 (90.0)	0.166	14 (73.7)	9 (47.4)	0.612	17 (100.0)	10 (76.9)	0.037^*^
No	7 (23.3)	3 (10.0)		5 (26.3)	2 (18.2)		0 (0.0)	3 (23.1)	
Phosphate binders									
Yes	27 (90.0)	28 (93.3)	0.640	16 (84.2)	11 (100.0)	0.165	15 (88.2)	13 (100.0)	0.201
No	3 (10.0)	2 (6.7)		3 (15.8)	0 (0.0)		2 (11.8)	0 (0.0)	
Supplemental iron									
Yes	26 (86.7)	25 (83.3)	0.718	16 (84.2)	10 (90.9)	0.603	14 (82.4)	11 (84.6)	0.869
No	4 (13.3)	5 (16.7)		3 (15.8)	1 (9.1)		3 (17.6)	2 (15.4)	
Subjective global assessment									
Normal	12 (44.0)	12 (40.0)	0.143	8 (42.1)	4 (36.4)	0.519	8 (47.1)	4 (30.8)	0.346
Mild malnourished	15 (50.0)	14 (46.7)		9 (47.4)	6 (54.6)		8 (47.1)	6 (46.2)	
Moderate to severe malnourished	0 (0)	4 (13.3)		0 (0)	0 (0)		1 (5.9)	3 (23.1)	
No response	3 (10.0)	0 (0)		2 (10.5)	1 (9.1)		0 (0)	0 (0)	
Anthropometry									
Body mass index (kg/m^2^)	23.08 ± 3.99	25.62 ± 4.24	0.020^*^	24.12 ± 4.02	21.28 ± 3.99	0.059	25.77 ± 4.56	25.45 ± 4.0	0.841
Mid-arm circumference (cm)	28.63 ± 5.01	28.45 ± 3.93	0.875	30.07 ± 5.28	26.27 ± 3.60	0.045^*^	27.68 ± 4.54	29.32 ± 3.04	0.250
Hand grip strength (kg)	20.63 ± 9.26	25.60 ± 8.09	0.034^*^	24.96 ± 8.60	13.93 ± 5.64	0.001^**^	30.04 ± 7.40	20.51 ± 5.60	0.000^***^
Pre-dialysis diet education									
Yes	27 (90.0)	30 (100.0)	0.206	16 (84.2)	11 (92.3)	0.165	17 (100.0)	13 (100.0)	NA
No	3 (10.0)	0 (0.0)		3 (15.8)	0 (0.0)		0 (0.0)	0 (0.0)	
Experience of dietary support programs									
Yes	1 (3.3)	0 (0.0)	0.313	1 (5.3)	0 (0.0)	0.439	0 (0.0)	0 (0.0)	NA
No	29 (96.7)	30 (100.0)		18 (94.7)	11 (100.0)		17 (100.0)	13 (100.0)	

Data are presented as Mean ± SD or N (%)

*NA* Not available due to small number of cases

* *p* < 0.05, ** *p* < 0.01, *** *p* < 0.001

### Dietary behaviors

The results of dietary behaviors, obtained using the self-report questionnaire, are shown in Table 2. There were significant differences between the two groups in 6 out of 10 items about eating habits. The frequency of eating three meals a day (Question No. 1) was significantly higher in the HD group than in the PD group; HD: 1.45 ± 0.62*,* PD: 0.82 ± 0.84 (*p* = 0.002). In addition, the frequency of intake of milk (Question No. 4), sugary food (Question No. 8–1), and fried food (Question No. 8–2); consideration of amount of drinking water (Question No. 9); and eating out (Question No. 10) was higher in the HD group than the PD group.

**Table 2:** Dietary behaviors of subjects

Variables	HD (n = 30)	PD (n = 30)	*p*-value	HD (n = 30)			PD (n = 30)		
				Male (n = 19)	Female (n = 11)	*p* -value	Male (*n* = 17)	Female (*n* = 13)	*p* -value
1. How many days per week do you have three meals a day?	1.45 ± 0.62	0.82 ± 0.84	0.002^*^	1.66 ± 0.53	1.09 ± 0.63	0.013^*^	1.21 ± 0.9	0.31 ± 0.44	< 0.001^**^
2. How many times per week do you have meals with a variety of grains fish, meat and vegetables?	1.07 ± 0.63	0.80 ± 0.64	0.108	1.05 ± 0.71	1.09 ± 0.49	0.875	0.94 ± 0.7	0.62 ± 0.55	0.170
3. How many times do you have fruits per week?	0.75 ± 0.67	0.85 ± 0.65	0.557	0.76 ± 0.77	0.73 ± 0.47	0.875	0.74 ± 0.7	1.00 ± 0.5	0.273
4. How many times do you have milk per week?	0.70 ± 0.78	0.27 ± 0.57	0.018^*^	0.74 ± 0.84	0.64 ± 0.71	0.741	0.24 ± 0.6	0.31 ± 0.59	0.736
5. Do you soak vegetables in water or boil them to remove potassium?	1.20 ± 0.78	0.78 ± 0.85	0.053	1.13 ± 0.93	1.32 ± 0.46	0.469	0.79 ± 0.9	0.77 ± 0.81	0.938
6. How many times per week do you have salted sea foods, pickled vegetables, ham, sausage canned fish, etc.?	1.77 ± 0.49	1.50 ± 0.74	0.106	1.68 ± 0.56	1.91 ± 0.3	0.163	1.32 ± 0.8	1.73 ± 0.59	0.139
7. How many days per week do you have 2 meals a day with a dish of protein?	0.68 ± 0.64	0.97 ± 0.56	0.071	0.58 ± 0.71	0.86 ± 0.45	0.192	1.27 ± 0.5	0.58 ± 0.4	< 0.001^**^
8–1. How many times per week do you have sugary foods for calorific replenishment?	0.88 ± 0.64	0.30 ± 0.41	< 0.001^**^	0.89 ± 0.74	0.86 ± 0.45	0.900	0.32 ± 0.4	0.27 ± 0.39	0.724
8–2. How many times per week do you have fried foods for calorific replenishment?	0.75 ± 0.58	0.25 ± 0.39	< 0.001^**^	0.74 ± 0.63	0.77 ± 0.52	0.874	0.27 ± 0.4	0.23 ± 0.39	0.817
9. Do you consider the amount of water needed to consume every day?	1.28 ± 0.68	0.87 ± 0.68	0.021^*^	1.26 ± 0.71	1.32 ± 0.64	0.835	0.88 ± 0.7	0.85 ± 0.69	0.888
10. How many times per week do you eat out?	1.58 ± 0.66	0.72 ± 0.81	< 0.001^**^	1.53 ± 0.72	1.68 ± 0.56	0.542	0.82 ± 0.9	0.58 ± 0.76	0.416

Data are presented as Mean ± SD

* *p* < 0.05, ** *p* < 0.001

### Comparisons of nutritional intake and nutritional intake-to-recommended allowance ratio according to dialysis modality

The results of the Semi-FFQ are shown in Table 3. Intake of energy, carbohydrates, fat, protein, dietary fiber, water, vitamin E, vitamin C, thiamin, riboflavin, niacin, vitamin B_6_, folic acid, pantothenic acid, calcium, phosphorus, sodium, and potassium was higher in HD patients than PD patients.

**Table 3:** Nutrition intake and intake-to-recommended allowance ratio according to dialysis modality

Variables	HD (n = 30)	PD (n = 30)	*p*-value	HD (n = 30)			PD (n = 30)		
				Male (n = 19)	Female (n = 11)	*p*-value	Male (n = 16)	Female (n = 14)	*p*-value
Energy (kcal)	1547.45 ± 380.83	1203.37 ± 387.78	0.001^**^	1647.29 ± 389.97	1375.00 ± 308.55	0.058	1319.21 ± 434.25	1070.98 ± 287.18	0.080
(%)	(79.8 ± 26.1)	(53.5 ± 19.6)	(0.000^***^)	(76.8 ± 28.4)	(85.0 ± 21.9)	(0.421)	(56.36 ± 23.24)	(50.22 ± 14.52)	(0.401)
Carbohydrate (g)	231.98 ± 55.40	176.87 ± 54.66	0.000^***^	245.53 ± 57.53	208.57 ± 44.63	0.078	193.38 ± 62.12	158.00 ± 38.66	0.076
(%)	(95.7 ± 31.2)	(63.1 ± 23.1)	(0.000^***^)	(91.6 ± 34.3)	(102.8 ± 25.0)	(0.356)	(66.35 ± 27.9)	(59.31 ± 16.16)	(0.399)
Fat (g)	40.97 ± 13.07	32.75 ± 12.71	0.016^*^	43.78 ± 13.74	36.13 ± 10.71	0.124	34.97 ± 13.22	30.21 ± 12.07	0.314
(%)	(65.4 ± 24.0)	(45.0 ± 18.7)	(0.001^**^)	(62.7 ± 24.4)	(69.9 ± 23.6)	(0.437)	(46.00 ± 19.71)	(43.82 ± 18.09)	(0.756)
Protein (g)	62.92 ± 25.39	50.60 ± 19.65	0.040^*^	67.50 ± 29.45	55.01 ± 14.11	0.199	56.41 ± 22.54	43.97 ± 13.66	0.084
(%)	(76.6 ± 20.6)	(81.7 ± 16.6)	(0.295)	(85.1 ± 20.4)	(61.8 ± 10.1)	(0.001^**^)	(87.72 ± 19.66)	(74.77 ± 8.49)	(0.026^*^)
Dietary Fiber (g)	15.92 ± 4.51	12.29 ± 3.98	0.002^*^	16.22 ± 4.91	15.41 ± 3.89	0.642	12.41 ± 4.0	12.15 ± 4.12	0.866
(%)	(63.7 ± 18.1)	(49.2 ± 15.9)	(0.002^**^)	(64.9 ± 19.7)	(61.6 ± 15.6)	(0.642)	(49.63 ± 15.93)	(48.6171 ± 16.49)	(0.866)
Water (ml)	687.80 ± 187.49	552.4 ± 176.31	0.006^**^	716.23 ± 197.47	638.70 ± 166.01	0.283	575.63 ± 182.22	526.05 ± 172.10	0.452
(%)	(45.9 ± 12.5)	(36.8 ± 11.8)	(0.006^**^)	(47.8 ± 13.2)	(42.6 ± 11.1)	(0.283)	(38.38 ± 12.15)	(35.07 ± 11.47)	(0.452)
Vitamin E (mg)	15.33 ± 4.57	10.7 ± 3.78	0.000^***^	15.57 ± 4.97	14.92 ± 3.98	0.710	11.20 ± 3.69	10.17 ± 3.95	0.467
(%)	(102.2 ± 30.5)	(71.5 ± 25.2)	(0.000^***^)	(103.8 ± 33.1)	(99.4 ± 26.5)	(0.710)	(74.67 ± 24.57)	(67.81 ± 26.36)	(0.467)
Vitamin C (mg)	89.86 ± 38.42	68.94 ± 38.66	0.040^*^	87.81 ± 44.17	93.41 ± 27.31	0.708	67.83 ± 33.32	70.21 ± 45.27	0.870
(%)	(99.9 ± 42.7)	(76.6 ± 43.0)	(0.040^*^)	(97.6 ± 49.1)	(103.8 ± 30.4)	(0.708)	(75.36 ± 37.02)	(78.01 ± 50.30)	(0.870)
Thiamin (mg)	0.97 ± 0.33	0.81 ± 0.28	0.046^*^	1.05 ± 0.37	0.83 ± 0.21	0.089	0.87 ± 0.31	0.73 ± 0.23	0.179
(%)	(80.7 ± 27.6)	(67.3 ± 23.3)	(0.046^*^)	(87.2 ± 30.6)	(69.4 ± 17.2)	(0.089)	(72.66 ± 25.93)	(61.07 ± 19.02)	(0.179)
Riboflavin (mg)	0.97 ± 0.28	0.79 ± 0.24	0.009^**^	1.00 ± 0.29	0.93 ± 0.26	0.535	0.85 ± 0.25	0.71 ± 0.22	0.130
(%)	(74.8 ± 21.3)	(60.7 ± 18.6)	(0.009^**^)	(76.7 ± 22.5)	(71.5 ± 19.7)	(0.535)	(65.58 ± 19.14)	(55.22 ± 16.96)	(0.130)
Niacin (mg)	13.31 ± 4.84	10.77 ± 3.62	0.025^*^	14.38 ± 5.52	11.45 ± 2.68	0.061	11.58 ± 3.68	9.84 ± 3.46	0.194
(%)	(83.2 ± 30.3)	(67.3 ± 22.7)	(0.025^*^)	(89.9 ± 34.5)	(71.5 ± 16.8)	(0.061)	(72.39 ± 22.98)	(61.51 ± 21.61)	(0.194)
Vitamin B_6_ (mg)	1.27 ± 0.4	1.03 ± 0.35	0.017^*^	1.36 ± 0.45	1.11 ± 0.25	0.061	1.08 ± 0.37	0.98 ± 0.32	0.437
(%)	(292.4 ± 166.0)	(230.7 ± 121.6)	(0.017^*^)	(282.0 ± 161.4)	(310.5 ± 180.0)	(0.658)	(100.11 ± 0.04)	(100.10 ± 0.032)	(0.437)
Folic acid (μg)	400.5 ± 102.59	311.18 ± 88.01	0.001^**^	399.71 ± 112.13	402.07 ± 88.8	0.953	324.78 ± 85.50	295.64 ± 91.41	0.375
(%)	(40.1 ± 10.3)	(31.1 ± 8.8)	(0.001^**^)	(40.0 ± 11.2)	(40.2 ± 8.9)	(0.953)	(32.48 ± 8.55)	(29.56 ± 9.14)	(0.375)
Vitamin B_12_ (μg)	7.02 ± 3.98	5.54 ± 2.92	0.106	6.77 ± 3.87	7.45 ± 4.32	0.658	6.29 ± 2.60	4.67 ± 3.11	0.132
(%)	(292.4 ± 166.0)	(230.7 ± 121.6)	(0.106)	(282.0 ± 161.4)	(310.5 ± 180.0)	(0.658)	(262.19 ± 108.49)	(194.70 ± 129.58)	(0.132)
Pantothenic Acid (mg)	4.33 ± 1.18	3.23 ± 0.89	0.000^***^	4.79 ± 1.08	3.54 ± 0.90	0.003^**^	3.58 ± 0.90	2.82 ± 0.71	0.017^*^
(%)	(86.6 ± 23.5)	(64.6 ± 17.8)	(0.000^***^)	(95.7 ± 21.7)	(70.7 ± 18.1)	(0.003^**^)	(71.68 ± 17.97)	(56.47 ± 14.14)	(0.017^*^)
Calcium (mg)	394.58 ± 128.42	307.87 ± 104.39	0.006^**^	384.39 ± 141.19	412.19 ± 106.79	0.577	320.06 ± 105.12	293.94 ± 105.67	0.504
(%)	(19.7 ± 6.4)	(15.4 ± 5.2)	(0.006^**^)	(19.2 ± 7.1)	(20.6 ± 5.3)	(0.577)	(16.11 ± 5.26)	(14.70 ± 5.28)	(0.504)
Phosphorus (mg)	840.45 ± 253.64	670.14 ± 220.90	0.007^**^	886.32 ± 283.3	761.20 ± 176.61	0.198	728.73 ± 239.68	603.18 ± 183.08	0.122
(%)	(105.1 ± 31.7)	(83.8 ± 27.6)	(0.007^**^)	(110.8 ± 35.4)	(95.2 ± 22.1)	(0.198)	(91.09 ± 29.96)	(75.40 ± 22.89)	(0.122)
Sodium (mg)	3581.06 ± 1235.23	2675.09 ± 818.76	0.002^**^	3559.27 ± 1257.9	3618.69 ± 1254.7	0.902	2985.25 ± 791.57	2320.62 ± 720.25	0.024^*^
(%)	(179.1 ± 61.8)	(133.8 ± 40.9)	(0.002^**^)	(178.0 ± 62.9)	(180.9 ± 62.7)	(0.902)	(149.26 ± 39.58)	(116.03 ± 36.01)	(0.024^*^)
Potassium (mg)	2100.44 ± 600.58	1602.47 ± 509.12	0.001^**^	2153.44 ± 668.77	2008.91 ± 475.85	0.497	1650.73 ± 546.05	1547.31 ± 477.54	0.588
(%)	(105.0 ± 30.0)	(80.1 ± 25.5)	(0.001^**^)	(107.7 ± 33.4)	(100.5 ± 23.8)	(0.497)	(82.54 ± 27.30)	(77.37 ± 23.88)	(0.588)
Cholesterol (mg)	337.25 ± 171.25	259.14 ± 130.88	0.052	375.13 ± 194.12	271.83 ± 98.9	0.113	307.02 ± 136.72	204.42 ± 102.97	0.030^*^
(%)	(169.5 ± 86.1)	(130.2 ± 65.8)	(0.052)	(188.5 ± 97.6)	(136.6 ± 49.7)	(0.113)	(154.28 ± 68.70)	(102.72 ± 51.74)	(0.030^*^)

Data are presented as Mean ± SD

Intake-to-recommended allowance ratio analysis are presented in parentheses below the nutrition intake

* *p* < 0.05, ** *p* < 0.01, *** *p* < 0.001

We also compared nutrient intake to recommended dietary allowance for dialysis patients [17, 18]. The intake of most of the major nutrients (i.e., energy, carbohydrates, fat, protein, dietary fiber, water, vitamin C, thiamine, riboflavin, niacin, folic acid, pantothenic acid, and calcium) was lower than the recommended amount in both the HD and PD groups. The nutrients with intakes over the recommended allowance were vitamin E, vitamin B_6_, vitamin B_12_, phosphorus, sodium, potassium, and cholesterol in the HD group and vitamin B_6,_ vitamin B_12_, sodium, and cholesterol in the PD group.

### Laboratory data

Data on serum albumin, BUN, creatinine, hemoglobin, hematocrit, sodium, phosphorus, calcium, potassium, and serum glucose are shown in Table 4. Serum albumin and potassium levels were significantly higher in HD patients than in PD patients. Except for serum albumin and potassium, there were no significant differences in the laboratory findings between the HD and PD groups.

**Table 4:** Comparison of laboratory findings

Variables	Reference range	HD (n = 30)	PD (n = 30)	*p*-value	HD (*n* = 30)			PD (n = 30)		
					Male (n = 19)	Female (n = 11)	*p*-value	Male (n = 16)	Female (n = 14)	*p* -value
Albumin (g/dL)	3.5~5.0	3.7 ± 0.4	3.4 ± 0.4	0.043^*^	3.6 ± 0.4	3.9 ± 0.2	0.032^*^	3.4 ± 0.5	3.5 ± 0.4	0.661
BUN (mg/dL)	10~20	64.9 ± 19.7	58.4 ± 16.5	0.169	62.1 ± 18.2	69.9 ± 22.0	0.299	65.3 ± 15.5	50.5 ± 14.3	0.011^*^
Creatinine (mg/dL)	0.5~1.2	12.7 ± 15.0	11.1 ± 3.5	0.561	14.5 ± 18.8	9.7 ± 2.4	0.412	12.3 ± 3.7	9.7 ± 2.7	0.034^*^
Hemoglobin (g/dL)	9~11.5	10.4 ± 1.0	10.4 ± 1.6	0.883	10.5 ± 1.0	10.3 ± 0.9	0.460	10.2 ± 1.9	10.6 ± 1.0	0.456
Hematocrit (%)	37~52	31.1 ± 2.9	30.9 ± 4.7	0.862	31.1 ± 2.9	31.1 ± 2.8	0.963	30.1 ± 6.0	31.8 ± 2.7	0.313
Sodium (mEq/L)	136~145	136.9 ± 3.1	137.0 ± 3.9	0.941	137.1 ± 3.0	136.7 ± 3.5	0.788	136.0 ± 4.0	138.2 ± 3.4	0.107
Phosphorus (mg/dL)	3.0~4.5	5.7 ± 1.9	5.8 ± 1.6	0.948	5.7 ± 2.0	5.8 ± 1.9	0.863	5.8 ± 1.7	5.7 ± 1.6	0.803
Calcium (mg/dL)	9.0~10.5	8.7 ± 0.9	8.6 ± 0.8	0.705	8.4 ± 0.8	9.1 ± 0.9	0.063	8.4 ± 0.9	8.8 ± 0.7	0.232
Potassium (mEq/L)	3.5~5.0	5.1 ± 0.7	4.4 ± 0.7	< 0.001^***^	5.0 ± 0.6	5.5 ± 0.8	0.050	4.4 ± 0.7	4.5 ± 0.8	0.886
Serum glucose (mg/dL)	70~110	162.1 ± 80.2	131.6 ± 95.1	0.185	151.8 ± 68.8	179.7 ± 98.0	0.368	121.7 ± 76.9	143.0 ± 114.3	0.549
Bicarbonate (mg/dL)	22~29	21.08 ± 3.11	26.23 ± 2.62	0.000^***^	21.6 ± 2.8	19.6 ± 3.6	0.137	25.3 ± 2.4	27.3 ± 2.5	0.037^*^

Data are presented as Mean ± SD

* *p* < 0.05, ** *p* < 0.01, *** *p* < 0.001

## Discussion

In this study, we compared the dietary behaviors and nutrient intake of prevalent HD and PD patients, and found that PD patients had worse dietary behaviors and lower dietary intake compared to HD patients by using semi-FFQ. There are several parameters that may be indicative of PEW in individuals with kidney disease, not only in dietary intake mentioned above, but also clinical, biochemical parameters. We analyzed SGA for nutritional scoring system; BMI for body mass; MAC, HGS for muscle mass; and laboratory marker. SGA is one of the well-established tools to assess nutritional status and a feasible method to ascertain PEW [19, 20]. The proportion of patients whose nutritional status deteriorated from well-nourished to malnourished or remained as malnourished for 1 year after the start of dialysis treatment was higher in the PD group than the HD group [11]. The changes in nutritional status assessed by SGA during the first year were associated with mortality in incident ESRD patients. In this study, PD patients had poorer dietary behaviors and subsequently less sufficient dietary intake compared to HD patients. There were four (13.3%) moderately to severely malnourished PD patients, while none of the HD patients was moderately to severely malnourished. It is associated with widespread malnutrition among PD patients, and it may be responsible for the overall higher mortality in PD patients than HD patients [21, 22]. BMI was higher in PD patients compared to the HD patients, which is a contradictory result to the worse nutrient intake in PD. Even though we excluded the participants with distinct edema observed by the investigators, BMI could be affected by fat mass or hydration status. We have to consider the possibility that PD patients have greater volume expansion than HD patients. In addition, HGS was higher in PD patients than in HD patients. The cut-off values of HGS in male and female in healthy Korean population (median age of 49.3) were 28.6 and 16.4 kg, respectively [23]. HGS was higher in PD patients compared to HD patients which are somewhat contrary result to worse nutrient intake and diet behavior in PD. We surmised that it was associated with the lower age of PD patients.

Next, we compared the dietary behaviors of the HD and PD groups. In the dietary behavior survey, HD patients scored higher than PD patients on most of the questions, which means that the HD group had better dietary behaviors than the PD group. Although HD patients tend to skip their meal on the day of HD, we need to pay attention to the lower rate of eating three meals a day in PD patients. Poor appetite, which is frequently seen in PD patients [24], may be one of the reasons for this. In addition, PD patients’ intake of sugary or fried foods, which are usually recommended for sufficient energy intake in dialysis patients, was less than that of HD patients. A recent study revealed that the nutritional status of HD and PD groups differs according to the dialysis vintage [25]. The dialysis duration < 2 years is associated with better hydration, nutritional state, and survival in PD patients, but longer dialysis duration reduces the benefits of the PD group. Dialysis vintage > 4 years is associated with similar hydration and mortality in both PD and HD groups. In this study, the mean duration of dialysis was 4–5 years in both groups. Therefore, considering the dialysis vintage of the patients included in this study, the higher proportion of malnourished patients in the PD group could have been expected.

We did analysis of dietary intake using the Semi-FFQ. HD group exhibited significantly higher consumption of dietary carbohydrates, fat, protein, and micronutrients than the PD group. A comparison of nutrient intake-to-recommended allowance ratio between the HD and PD groups [26–29] revealed that the HD group showed higher nutrient intake than the PD group. However, considering energy intake from dialysate glucose [30], it is likely that the total energy intake of the PD group was similar to that of the HD group. A previous study comparing the nutritional status of HD and PD groups in Korea suggested that the HD group became malnourished due to a lack of energy intake and the PD group developed malnutrition due to a lack of protein intake [31]. In other words, intraperitoneal glucose absorption in dialysis fluid provides energy supplementation, which was 364.44 ± 154.49 kcal/d of energy in this research, but loss of protein through peritoneal fluid is more crucial for the development of malnutrition in PD patients. The energy intake-to recommended allowance ratio in both HD and PD patients was low, and it was more prominent in the PD group than the HD group.

We also analyzed the laboratory parameters to assess nutrient status indirectly. Serum albumin levels were significantly higher in the HD group than the PD group, which is consistent with the results of previous studies [32]. The serum albumin value is considered a biomarker of visceral protein and a fundamental parameter of nutritional assessment [33]. We suggest that one of the reasons for the low serum albumin levels in the PD group is the significantly lower protein intake, which was revealed from the Semi-FFQ, and protein loss via PD fluid. Recent studies show that a low serum albumin level rather reflects a state of persistent inflammation and has limited value as a marker of nutritional status only [34]. Therefore, efforts to not only increase dietary protein intake but also reduce systemic inflammation are needed to increase serum albumin levels in PD patients. In this study, serum potassium levels were significantly lower in the PD group than the HD group. The lower level of serum potassium in PD patients may be attributed to their low potassium intake reported in the Semi-FFQ. A recent study suggested that low serum potassium is an independent risk factor for mortality in dialysis patients, and the major cause of death in PD patients with lower potassium was cardiovascular death and infection [35].

PEW is associated with mortality in patients with ESRD on dialysis [36], and this study also confirmed poor energy consumption by both HD and PD patients. In non-dialysis CKD patients, a neutral or slightly positive nitrogen balance can be maintained with a low-protein (0.6–0.8 g/kg/day) diet and restricted intake of sodium, potassium, and phosphorus [31, 32]. In patients on maintenance HD and PD, however, their protein requirement is as high as 1.2–1.3 g/kg/day [26] for the compensation of dialysis-related protein loss, extra energy expenditure, and persistent inflammation [33]. In this study, neither HD nor PD patients consumed adequate amounts of energy and protein compared to the recommended allowance, and it was more prominent in the PD group than the HD group. It should receive attention to ensure better outcomes and maintain high quality of life.

This study has several limitations. First, we did not use a diet diary for a complete nutrient assay. Although the Semi-FFQ, which was used in this study, was validated elsewhere [15], there were several limitation of questionnaire itself. FFQ can be underestimated because of the inadequate coverage of all available food items to an individual. Since we intended to compare the nutritional intake between PD and HD, this limitation would not have a significant effect on the outcome of the original question. Second, we did not evaluate the nitrogen balance or precise inflammatory status of subjects. Third, this was a cross-sectional study, and the participants were not followed. Clinical effects of dietary behaviors and nutrient intake could not be investigated due to the cross-sectional study design. Finally, a small number of patients were included in this study. FFQ is favored for large scale epidemiologic study because of FFQ’s lack of accuracy to amount dietary intakes to an individual level. However, we intended to compare essential nutrient element to HD with PD groups, not to evaluate absolute intake. Although statistical power may be low, we believe FFQ may be a useful tool in comparing nutrient intake related to dialysis outcome in ESRD patients.

## Conclusion

Even though there were several studies that have compared the nutritional status of HD with PD patients, there was no study that has assessed the nutrient intake by the Semi-FFQ and dietary behavior according to dialysis modality. According to this study, nutritional status, dietary behaviors, and nutrient intake-to-recommended allowance ratio were worse in PD patients compared to HD patients. It implies more intensive nutritional intervention may be needed for PD patients. Through this, we can use semi-FFQ as a valuable tool to figure out patients who ingest higher or lower amount of essential nutrient in dialysis patients. We also have to understand multiple factors contributing to PEW of dialysis patients, and individualized therapeutic approach is needed.

## Supplementary information

**Additional file 1: Table S1.** Semi-quantitative Food Frequency Questionnaire. **Table S2.** Dialysis modality.

## Data Availability

The dataset used in the analysis is available with the corresponding author and will be released on request.

## Abbreviations

BMI: Body mass index
BUN: Blood urea nitrogen
CKD: Chronic kidney disease
ESRD: End-stage renal disease
HD: Hemodialysis
HGS: Hand grip strength
MAC: Mid-arm circumference
PD: Peritoneal dialysis
PEW: Protein-energy wasting
Semi-FFQ: Semi-quantitative Food Frequency Questionnaire
SGA: Subjective global assessment
