# Supplementary material for: Comparison of hemodialysis and peritoneal dialysis patients’ dietary behaviors
Source: BMC Nephrol. 2020 Mar 10;21:91. doi: 10.1186/s12882-020-01744-6 (PMC7063748; doi:10.1186/s12882-020-01744-6)
Supplement: Supplementary file 1 — Additional file 1: Table S1. Semi-quantitative Food Frequency Questionnaire. Table S2. Dialysis modality. [file 12882_2020_1744_MOESM1_ESM.docx]

| Intake  frequency    Food item | Never/  Rarely | Times per month | | Times per week | | | Times per day | | | Average intake of one serving: | | | | | |
| --- | --- | --- | --- | --- | --- | --- | --- | --- | --- | --- | --- | --- | --- | --- | --- |
|  |  | 1 | 2~3 | 1 | 2~4 | 5~6 | 1 | 2 | 3 |  |  |  |  |  |  |
| 1. White rice | ① | ② | ③ | ④ | ⑤ | ⑥ | ⑦ | ⑧ | ⑨ | 1 Bowl (300ml) | | | | | |
|  |  |  |  |  |  |  |  |  |  | ①  **½** | ②  **1** | | ③  **1½** | | ④  **2** |
| 2. Mixed rice  (Bean rice, barely rice) | ① | ② | ③ | ④ | ⑤ | ⑥ | ⑦ | ⑧ | ⑨ | 1 Bowel (300ml) | | | | | |
|  |  |  |  |  |  |  |  |  |  | ①  **½** | ②  **1** | | ③  **1½** | | ④  **2** |
| 3.Bibimbap, Gim-bap, Fried rice | ① | ② | ③ | ④ | ⑤ | ⑥ | ⑦ | ⑧ | ⑨ | 1 Serving (500ml) | | | | | |
|  |  |  |  |  |  |  |  |  |  | ①  **½** | | ②  **1** | | ③ **1½** | |
| 4. Ramen, Instant ramen | ① | ② | ③ | ④ | ⑤ | ⑥ | ⑦ | ⑧ | ⑨ | 1 Serving | | | | | |
|  |  |  |  |  |  |  |  |  |  | ①  **½** | | ②  **1** | | ③  **1½** | |
| 5. Noodle  (Cold noodle, Kalguksu, U-don) | ① | ② | ③ | ④ | ⑤ | ⑥ | ⑦ | ⑧ | ⑨ | 1 Serving (=1000ml) | | | | | |
|  |  |  |  |  |  |  |  |  |  | ①  **½** | | ②  **1** | | ③  **1½** | |
| 6. Bread  (Loaf bread, Red bean bread, Steamed bun, Cream bread, Cake) | ① | ② | ③ | ④ | ⑤ | ⑥ | ⑦ | ⑧ | ⑨ | Two white loaves,  A piece of cake | | | | | |
|  |  |  |  |  |  |  |  |  |  | ①  **½** | | ②  **1** | | ③  **1½** | |
| 7. Rice cake  (Steamed white rice cake, Injeolmi, Topokki) | ① | ② | ③ | ④ | ⑤ | ⑥ | ⑦ | ⑧ | ⑨ | Half of steamed white rice cake or one cup of Topokki (200ml) | | | | | |
|  |  |  |  |  |  |  |  |  |  | ①  **½** | | ②  **1** | | ③  **1½** | |
| 8. Cereal | ① | ② | ③ | ④ | ⑤ | ⑥ | ⑦ | ⑧ | ⑨ | 1 Bowl of cereal (250ml, , including milk) | | | | | |
|  |  |  |  |  |  |  |  |  |  | ①  **½** | | ②  **1** | | ③  **1½** | |
| 9. Pork  (Bacon, boiled pork splice, Barbequed pork) | ① | ② | ③ | ④ | ⑤ | ⑥ | ⑦ | ⑧ | ⑨ | 1 Serving (150g=1cup) | | | | | |
|  |  |  |  |  |  |  |  |  |  | ①  **½** | | ②  **1** | | ③  **1½** | |
| 10. Beef  ( Barbequed beef, Roasted beef, Galbi-jjim) | ① | ② | ③ | ④ | ⑤ | ⑥ | ⑦ | ⑧ | ⑨ | 1 Serving (1 Cup, 150g) | | | | | |
|  |  |  |  |  |  |  |  |  |  | ①  **½** | | ②  **1** | | ③  **1½** | |
| 11. Beef soup | ① | ② | ③ | ④ | ⑤ | ⑥ | ⑦ | ⑧ | ⑨ | 1 Serving (20 g) | | | | | |
|  |  |  |  |  |  |  |  |  |  | ①  **½** | | ②  **1** | | ③  **1½** | |

Table S1. Semi-quantitative Food Frequency Questionnaire

Please tell us how often you have eaten each of the following foods in the past year, and how often you have eaten on average once.

| Intake  frequency    Food item | Never/  Rarely | Times per month | | Times per week | | | Times per day | | | Average intake of one serving: | | |
| --- | --- | --- | --- | --- | --- | --- | --- | --- | --- | --- | --- | --- |
|  |  | 1 | 2~3 | 1 | 2~4 | 5~6 | 1 | 2 | 3 |  |  |  |
| 12. Ham, Sausage | ① | ② | ③ | ④ | ⑤ | ⑥ | ⑦ | ⑧ | ⑨ | ¼ Cup (50ml) or 1 Piece | | |
|  |  |  |  |  |  |  |  |  |  | ①  **⅛** | ②  **1** | ③  **2** |
| 13. Chicken  (Boiled chicken, steamed chicken) | ① | ② | ③ | ④ | ⑤ | ⑥ | ⑦ | ⑧ | ⑨ | 1 Serving (800ml) | | |
|  |  |  |  |  |  |  |  |  |  | ①  ½ | ②  1 | ③  1½ |
| 14. Fried chicken | ① | ② | ③ | ④ | ⑤ | ⑥ | ⑦ | ⑧ | ⑨ | 2 Pieces of drumstick  (400ml) or edible portion  (200ml) | | |
|  |  |  |  |  |  |  |  |  |  | ①  ½ | ②  1 | ③  1½ |
| 15. Duck meat | ① | ② | ③ | ④ | ⑤ | ⑥ | ⑦ | ⑧ | ⑨ | 1Cup, 150g | | |
|  |  |  |  |  |  |  |  |  |  | ①  ½ | ②  1 | ③  1½ |
| 16. Fish  (Cutlass fish, Mackerel, Saury, Gizzard shad, Cero) | ① | ② | ③ | ④ | ⑤ | ⑥ | ⑦ | ⑧ | ⑨ | ¼ Cup (1cut , 50ml) | | |
|  |  |  |  |  |  |  |  |  |  | ①  ½ | ②  1 | ③  1½ |
| 17. Anchovy,  Fried anchovy | ① | ② | ③ | ④ | ⑤ | ⑥ | ⑦ | ⑧ | ⑨ | 1TS (15ml) | | |
|  |  |  |  |  |  |  |  |  |  | ①  1  ts | ②  1  TS | ③  ¼  C |
| 18. Squid | ① | ② | ③ | ④ | ⑤ | ⑥ | ⑦ | ⑧ | ⑨ | ¼ Squid (½ Cup, 100ml) | | |
|  |  |  |  |  |  |  |  |  |  | ①  ⅛ | ②  ¼ | ③  ½ |
| 19. Pickled seafood  (Salted shrimp, Squid, Clams) | ① | ② | ③ | ④ | ⑤ | ⑥ | ⑦ | ⑧ | ⑨ | 1ts (5ml) | | |
|  |  |  |  |  |  |  |  |  |  | ①  ½ | ②  1 | ③  1½ |
| 20. Fish cake  (Soup, Fried) | ① | ② | ③ | ④ | ⑤ | ⑥ | ⑦ | ⑧ | ⑨ | ½ Cup (100ml) or  Soup (250ml) | | |
|  |  |  |  |  |  |  |  |  |  | ①  ¼ | ②  ½ | ③  1 |
| 21. Tofu | ① | ② | ③ | ④ | ⑤ | ⑥ | ⑦ | ⑧ | ⑨ | 1/4 Block (120g) | | |
|  |  |  |  |  |  |  |  |  |  | ①  ¼ | ②  ½ | ③  1 |
| 22. Soy bean (boiled) | ① | ② | ③ | ④ | ⑤ | ⑥ | ⑦ | ⑧ | ⑨ | 2TS (20g) | | |
|  |  |  |  |  |  |  |  |  |  | ①  ½ | ②  1 | ③  1½ |
| 23. Fried egg, Egg roll | ① | ② | ③ | ④ | ⑤ | ⑥ | ⑦ | ⑧ | ⑨ | 1 Fried egg or  4 Pieces of egg roll | | |
|  |  |  |  |  |  |  |  |  |  | ①  ½ | ②  1 | ③  1½ |

| Intake  frequency    Food item | Never/  Rarely | Times per month | | Times per week | | | Times per day | | | Average intake of one serving: | | |
| --- | --- | --- | --- | --- | --- | --- | --- | --- | --- | --- | --- | --- |
|  |  | 1 | 2~3 | 1 | 2~4 | 5~6 | 1 | 2 | 3 |  |  |  |
| 24. Boiled egg, Steamed egg | ① | ② | ③ | ④ | ⑤ | ⑥ | ⑦ | ⑧ | ⑨ | 1 Boiled egg or  1/2 Cup of steamed egg | | |
|  |  |  |  |  |  |  |  |  |  | ①  ½ | ②  1 | ③  1½ |
| 25. Low level of potassium contained vegetable  ;Cabbage, Perilla leaves, Cucumbers, Bean sprouts, etc. | ① | ② | ③ | ④ | ⑤ | ⑥ | ⑦ | ⑧ | ⑨ | ¼ Cup (50ml) or  Soup (250ml) | | |
|  |  |  |  |  |  |  |  |  |  | ①  ⅛ | ②  ¼ | ③  ½ |
| 26. Medium level of potassium contained vegetable  ; Lettuce, Pulley, Hot radish, Zucchini, etc. | ① | ② | ③ | ④ | ⑤ | ⑥ | ⑦ | ⑧ | ⑨ | ¼ Cup (50ml) | | |
|  |  |  |  |  |  |  |  |  |  | ①  ⅛ | ②  ¼ | ③  ½ |
| 27. High level of potassium contained vegetable  ; Ah-wuk, beet, water parsley, chives, spinach, etc. | ① | ② | ③ | ④ | ⑤ | ⑥ | ⑦ | ⑧ | ⑨ | ¼ Cup (50ml) | | |
|  |  |  |  |  |  |  |  |  |  | ①  ⅛ | ②  ¼ | ③  ½ |
| 28. Kimchi | ① | ② | ③ | ④ | ⑤ | ⑥ | ⑦ | ⑧ | ⑨ | ¼ Cup (50ml) | | |
|  |  |  |  |  |  |  |  |  |  | ①  ⅛ | ②  ¼ | ③  ½ |
| 29. Mushroom | ① | ② | ③ | ④ | ⑤ | ⑥ | ⑦ | ⑧ | ⑨ | ¼ Cup (50ml) | | |
|  |  |  |  |  |  |  |  |  |  | ①  ⅛ | ②  ¼ | ③  ½ |
| 30. Steamed potato, Fried potato, Boiled potato | ① | ② | ③ | ④ | ⑤ | ⑥ | ⑦ | ⑧ | ⑨ | 1 Piece | | |
|  |  |  |  |  |  |  |  |  |  | ①  ½ | ②  1 | ③  2 |
| 31. Steamed sweet potato, Roasted sweet potato | ① | ② | ③ | ④ | ⑤ | ⑥ | ⑦ | ⑧ | ⑨ | 1 Piece | | |
|  |  |  |  |  |  |  |  |  |  | ①  ½ | ②  1 | ③  2 |
| 32. Steamed corn, Roasted corn | ① | ② | ③ | ④ | ⑤ | ⑥ | ⑦ | ⑧ | ⑨ | 1 Piece | | |
|  |  |  |  |  |  |  |  |  |  | ①  ½ | ②  1 | ③  1½ |
| 33. Milk | ① | ② | ③ | ④ | ⑤ | ⑥ | ⑦ | ⑧ | ⑨ | 1 Cup (200ml) | | |
|  |  |  |  |  |  |  |  |  |  | ①  ½ | ②  1 | ③  1½ |
| 34. Yogurt | ① | ② | ③ | ④ | ⑤ | ⑥ | ⑦ | ⑧ | ⑨ | 1 Cup (100g) | | |
|  |  |  |  |  |  |  |  |  |  | ①  ½ | ②  1 | ③  2 |
| 35. Soymilk | ① | ② | ③ | ④ | ⑤ | ⑥ | ⑦ | ⑧ | ⑨ | 1 Cup (200ml) | | |
|  |  |  |  |  |  |  |  |  |  | ①  ½ | ②  1 | ③  1½ |

| Intake  frequency    Food item | Never/  Rarely | Times per month | | Times per week | | | Times per day | | | Average intake of one serving: | | |  |  |  |  |
| --- | --- | --- | --- | --- | --- | --- | --- | --- | --- | --- | --- | --- | --- | --- | --- | --- |
|  |  | 1 | 2~3 | 1 | 2~4 | 5~6 | 1 | 2 | 3 |  |  |  |  |  |  |  |
| 36. Strawberry | ① | ② | ③ | ④ | ⑤ | ⑥ | ⑦ | ⑧ | ⑨ | 10 Piece or ⅔ Cup of juice | | |  | | |  |
|  |  |  |  |  |  |  |  |  |  | ①  5 | ②  10 | ③  15 |  |  |  |  |
| 37. Tomato, Cherry tomato | ① | ② | ③ | ④ | ⑤ | ⑥ | ⑦ | ⑧ | ⑨ | 1 Piece of tomato, 30 Pieces of cherry tomato, or  1 Cup of juice | | |  | | |  |
|  |  |  |  |  |  |  |  |  |  | ①  ½ | ②  1 | ③  2 |  |  |  |  |
| 38. Low level of potassium contained fruits; Persimmon, Apple, Plum , Grape Pineapple, etc. | ① | ② | ③ | ④ | ⑤ | ⑥ | ⑦ | ⑧ | ⑨ | 1 Piece (100ml) | | |  | | |  |
|  |  |  |  |  |  |  |  |  |  | ①  ⅓ | ②  1 | ③  2 |  |  |  |  |
| 39. Medium level of potassium contained fruits; Tangerines, Peaches, Pears, Strawberries, Oranges Watermelons, etc. | ① | ② | ③ | ④ | ⑤ | ⑥ | ⑦ | ⑧ | ⑨ | 1 Piece(100ml) | | |  | | |  |
|  |  |  |  |  |  |  |  |  |  | ①  ⅓ | ②  1 | ③  2 |  |  |  |  |
| 40. High level of potassium contained fruits; Banana, Melon, Kiwi, Tomato | ① | ② | ③ | ④ | ⑤ | ⑥ | ⑦ | ⑧ | ⑨ | 1 Piece(100ml) | | |  | | |  |
|  |  |  |  |  |  |  |  |  |  | ①  ½ | ②  1 | ③  2 |  |  |  |  |
| 41. Green Tea | ① | ② | ③ | ④ | ⑤ | ⑥ | ⑦ | ⑧ | ⑨ | 1 Cup (200ml) | | |  |  |  |  |
|  |  |  |  |  |  |  |  |  |  | ①  ½ | ②  1 | ③  1½ |  |  |  |  |
| 42. Soda | ① | ② | ③ | ④ | ⑤ | ⑥ | ⑦ | ⑧ | ⑨ | 1 Cup (200ml) | | |  |  |  |  |
|  |  |  |  |  |  |  |  |  |  | ①  ½ | ②  1 | ③  1½ |  |  |  |  |
| 43. Coffee | ① | ② | ③ | ④ | ⑤ | ⑥ | ⑦ | ⑧ | ⑨ | 1 Cup (200ml) | | |  |  |  |  |
|  |  |  |  |  |  |  |  |  |  | ①  ½ | ②  1 | ③  1½ |  |  |  |  |
| In addition to the above foods, please write down the type and frequency of your intake of the food that you usually eat frequently. | | | | | | | | | | | | |  |  |  |  |
| 44. Chinese Food | ① | ② | ③ | ④ | ⑤ | ⑥ | ⑦ | ⑧ | ⑨ |  | | |  |  |  |  |
| 45. Seaweeds | ① | ② | ③ | ④ | ⑤ | ⑥ | ⑦ | ⑧ | ⑨ |  | | |  |  |  |  |
| 46. Alcohol | ① | ② | ③ | ④ | ⑤ | ⑥ | ⑦ | ⑧ | ⑨ |  | | |  |  |  |  |
| 47. Extra | ① | ② | ③ | ④ | ⑤ | ⑥ | ⑦ | ⑧ | ⑨ |  | | |  |  |  |  |

Abbreviation: TS; Tablespoon, ts; teaspoon

Table S2. Dialysis modality

|  | Modality | Method | Patients (%) |
| --- | --- | --- | --- |
| HD | Hemodialysis | High flow | 6 (20.0%) |
|  |  | Low flow | 24 (80.0%) |
|  | Hemodiafilteration |  | 0 (0%) |
| PD | Continuous ambulatory peritoneal dialysis |  | 20 (66.7%) |
|  | Automated peritoneal dialysis | Continuous cyclic peritoneal dialysis | 6 (20.0%) |
|  |  | Nightly peritoneal dialysis | 4 (13.3%) |
|  | Peritoneal Equilibrium Test* | High | 0 (0%) |
|  |  | High Average | 14 (46.7%) |
|  |  | Low Average | 13 (43.3%) |
|  |  | Low | 3 (10%) |

PET transport classification: High, >0.83; high average, 0.65-0.82; low average, 0.5-0.65 ; low, <0.5
